# Secular Trends in Growth and Nutritional Status of Mozambican School-Aged Children and Adolescents

**DOI:** 10.1371/journal.pone.0114068

**Published:** 2014-12-04

**Authors:** Fernanda Karina dos Santos, José A. R. Maia, Thayse Natacha Q. F. Gomes, Timóteo Daca, Aspacia Madeira, Peter T. Katzmarzyk, António Prista

**Affiliations:** 1 CIFI2D, Kinanthropometry Lab, Faculty of Sport, University of Porto, Porto, Portugal; 2 CAPES Foundation, Ministry of Education of Brazil, Brasília – DF, Brazil; 3 Faculty of Physical Education and Sports, Pedagogical University, Maputo, Mozambique; 4 Pennington Biomedical Research Center, Louisiana State University System, Baton Rouge, Louisiana, United States of America; TNO, Netherlands

## Abstract

**Objectives:**

The purpose of this study was to examine secular changes in growth and nutritional status of Mozambican children and adolescents between 1992, 1999 and 2012.

**Methods:**

3374 subjects (1600 boys, 1774 girls), distributed across the three time points (523 subjects in 1992; 1565 in 1999; and 1286 in 2012), were studied. Height and weight were measured, BMI was computed, and WHO cut-points were used to define nutritional status. ANCOVA models were used to compare height, weight and BMI across study years; chi-square was used to determine differences in the nutritional status prevalence across the years.

**Results:**

Significant differences for boys were found for height and weight (p<0.05) across the three time points, where those from 2012 were the heaviest, but those in 1999 were the tallest, and for BMI the highest value was observed in 2012 (1992<2012, 1999<2012). Among girls, those from 1999 were the tallest (1992<1999, 1999>2012), and those from 2012 had the highest BMI (1999<2012). In general, similar patterns were observed when mean values were analyzed by age. A positive trend was observed for overweight and obesity prevalences, whereas a negative trend emerged for wasting, stunting-wasting (in boys), and normal-weight (in girls); no clear trend was evident for stunting.

**Conclusion:**

Significant positive changes in growth and nutritional status were observed among Mozambican youth from 1992 to 2012, which are associated with economic, social and cultural transitional processes, expressing a dual burden in this population, with reduction in malnourished youth in association with an increase in the prevalence of overweight and obesity.

## Introduction

It is generally accepted that human growth indicators are suitable markers of population health and nutritional status [1]–[3]. Although human growth is governed to some extent by genetic factors [4], [5], the genetic potential will be expressed if socioeconomic and environmental conditions, namely adequate nutrition, absence of infectious diseases, quality health care access, and availability of safe water, just to name a few, allows it, and more so in developing countries [6].

Childhood malnutrition is a long-standing and enduring public health problem in many developing countries [7], leading to wasting or stunting conditions and their combined effects (stunting and wasting). Wasting arises with children having low weight for their height, while stunting often results when infants and young children are exposed to under-nutrition, with growth failure beginning at approximately 3 months of age and continuing into the third year of life, resulting in a short stature for age [8]. Although global data show that the prevalence of childhood stunting and wasting has declined [7], these unfortunate conditions still present public health challenges, especially in developing countries. For example, globally, in 2012, 15.1% of children under 5 years of age were underweight, 24.7% were stunted, 8% were wasted (3% severely); further, 92% of stunted children, 96% of underweight children, and 97% of wasted children live in Africa and Asia [9].

Notwithstanding the continuing problem of under-nutrition in several regions, a worldwide increase in the prevalence of pediatric overweight and obesity is generally observed [10]. Remarkably, the fastest growth rates in the prevalence of overweight and obesity are observed in Africa and Asia [10], which is hypothesized to be related to the fast societal transitions observed in the last decades, with the amplified adoption of urban lifestyles characterized by abrupt changes in dietary patterns and physical activity levels [11].

In the last century, urbanization allowed for worldwide improvements in nutritional and health status, resulting in increased heights and weights in developed and developing countries (i.e., a positive secular trend) [4]. However, these positive changes varied between populations. For example, although many European countries observed a general mean height increase [12]–[14], German and Polish populations seemed to reach a plateau [15], [16]; on the contrary, in some African countries, no increase, or even a decrease in height was noted [17], along with positive secular trends observed in Kenya [17], Senegal [17], and Seychelles [18]. On the other hand, the global increasing rates of overweight and obesity shows a systematic positive secular trend in children and adults [10], [19].

Information regarding the impact of socioeconomic transformations on growth changes among children in developing countries [17], [20], [21], especially in Africa, is still scarce. Since it is clear that information on growth parameters provide quality indicators of health status [2], temporal growth studies may provide an understanding of the social, economic and cultural changes occurring in transitional periods.

In the last twenty years, since the end of the War, Mozambique has undergone an epidemiological and public health transition period due to positive changes in social and economic factors, which has had a positive impact in the quality of life and well-being of its population [22]. On the other hand, one of the burdens from this transition is the adoption of westernized ways of life with the proliferation of fast-food consumption, reduction of physical activity levels, and increased dissemination and use of technology at leisure and work, mainly in big cities such as the capital city of Maputo [23]. Thus, the purpose of this study is to examine the secular changes in growth and nutritional status of Mozambican children and adolescents, between 1992 (just after the War), 1999 (time of peace) and 2012 (period of economic growth).

## Methods

### Mozambique

Mozambique is a country located in Southeast Africa, bordered by the Indian Ocean, Tanzania, Malawi, Zambia, Zimbabwe, Swaziland, and South Africa. With an estimated total population (in 2012) of approximately 25 million [24], and a population growth rate of 2.4% [25], Mozambique has experienced significant economic growth over the last 20 years following the end of the War. Table 1 summarizes observed changes in socio-demographic and health indicators in Mozambique from the 1990’s to the 2010’s.

**Table 1:** Socio-demographic and health information from the 1990’s to the 2010’s for Mozambique [25], [39]–[45].

	Early 1990’s	Late 1990’s/Early 2000’s	2010’s
GNI/per capita ($)	87	260	510
HDI	0.281	0.352	0.327
Population lived in poverty (%)	>50	54.1	59.6
Life expectancy (years)	43	46.7	53
Urban population (%)	23	29	31.4
Child mortality (per 1000 live births)	226	172	84
Adult literacy (%)	39.5	46.7	50.6

Legend: HDI: Human Development Index; GNI: Gross National Income.

### Sample

The present study is part of the “Human Biological Variability - Implications for Physical Education, Sports, Preventive Medicine and Public Health” research project [26]. Briefly, its aims are to describe the patterns of human variability in growth, biological maturation and development of Mozambican youth, and to understand the role of genetic and environmental factors in the variability of these indicators in this population. Data collection occurred in three periods: 1992, 1999 and 2012.

The sample comprises children and adolescents aged 8–15 years, assessed in each time period. The sampling design, three-stage cluster sampling (areas, schools and students) has been consistent, as children and adolescents were enlisted from the same schools living in Maputo urban or suburban areas [27]. All children attending these schools were invited to take part in the project and consent forms, signed by parents or legal guardians were required. Children without a signed consent form, with chronic diseases, physical handicaps or psychological disorders, or younger than 8 years or older than 15 years, were excluded during sample selection and/or data screening. A total of 536 subjects with missing information, at random, were excluded during data analysis, and the final sample was distributed as follows: 523 subjects in 1992 (247 boys, 276 girls); 1565 subjects in 1999 (739 boys, 826 girls) and 1286 subjects in 2012 (614 boys, 672 girls). The total sample comprises 3374 subjects (1600 boys, 1774 girls; Table 2). The study protocol was approved by the Mozambican National Bioethics Committee.

**Table 2:** Sample size of each age and sex group according to the time period (B = boys; G = girls).

Age	1992			1999			2012			TOTAL
	B	G	Total	B	G	Total	B	G	Total	
8	27	34	61	52	66	118	68	77	145	324
9	22	35	57	65	84	149	76	101	177	383
10	38	32	70	53	95	148	105	110	215	433
11	37	42	79	65	77	142	72	88	160	381
12	35	27	62	123	97	220	84	85	169	451
13	41	41	82	155	163	318	64	50	114	514
14	19	37	56	138	111	249	94	120	214	519
15	28	28	56	88	133	221	51	41	92	369
**TOTAL**	**247**	**276**	**523**	**739**	**826**	**1565**	**614**	**672**	**1286**	**3374**

### Anthropometry

The same measurement protocol, as described by Lohman et al [28], was used in the three data collection periods. Height and weight were measured by the same trained personnel in order to minimize measurement errors and guarantee privacy: girls were measured by female technicians, and boys were measured by male technicians. Height was measured with a Harpender stadiometer (±0.1 cm; Holtain, Crymych, UK) with the head positioned in the Frankfurt plane, and a Seca scale (±0.1 kg; Seca, Germany) was used to measure weight. Subjects were assessed without shoes and naked in two private rooms, one for each sex. The body mass index (BMI) was calculated from measured height and weight (kg/m^2^).

### Biological Maturation

Biological maturity was assessed using procedures described by Tanner and Whitehouse [29], with children being classified according to their secondary sexual characteristics (pubic hair stages). Trained observers from the same sex rated all children/adolescents in a private room.

### Nutritional status

Nutritional status was determined according to cut-offs suggested by the World Health Organization (WHO) expert committee [3]. Z-scores for height and BMI percentiles were calculated relative to reference values of the WHO growth charts [30]. Children were classified into six groups: (1) normal weight and height (height for age ≥−2 SD and P5≤ BMI for age <P85), (2) low height-for-age (stunted - height for age <−2 SD), (3) low weight-for-height (wasted - BMI for age <P5), (4) low height-for-age and low weight-for-height (stunted and wasted - height for age <−2 SD and BMI for age <P5), (5) overweight (BMI for age ≥P85 and <P95), and (6) obese (BMI for age ≥P95).

During the nutritional status classification process only 12 cases were found to be overlapping (4 stunting-overweight and 8 stunting-obese). Since in the African context the prevalence of stunting is considerably higher and more prominent in public health terms than the prevalence of overweight and obesity, mainly due to highly adverse environmental constraints, we chose to classify these subjects as stunted. Their effects in the analysis were insignificant.

### Statistical analysis

Basic descriptive statistics were computed. Since a general positive trend for biological maturation was found along the years (data not sown), analysis of covariance (ANCOVA), controlling for age and biological maturity (entered as a continuous variable), was used to compare height, weight and BMI among years by sex. Furthermore, ANCOVA, adjusted for biological maturity, across the years was used to determine mean differences between years of evaluation, by age and sex, for each variable. Bonferroni adjustments were made in all comparisons. Differences in nutritional statuses’ prevalences across the years were tested by a chi-square test. SPSS 20.0 and WINPEPI were used in all analysis.

## Results

Results for the ANCOVA (mean±standard error, F and p-values) for height, weight and BMI by sex, controlling for age and biological maturation, are presented in Table 3. Statistically significant differences across time were observed in all variables: for boys, differences in height and weight (p<0.05) were observed in all three comparisons (1992–1999; 1992–2012; 1999–2012), where those from 2012 were the heaviest, but those in 1999 were the tallest. For BMI, the highest value was observed in 2012, with increases found between 1992–2012 and 1999–2012, but no difference between 1992 and 1999. Among girls, significant differences were observed in height and BMI, where those from 1999 were taller when compared to those from 1992 and 2012 (no significant difference was observed between 1992 and 2012); those from 2012 had higher BMI value than their counterparts from 1999, with no significant differences observed between 1992–1999 and 1992–2012; no significant differences were observed in weight.

**Table 3:** Age and biological maturation-adjusted means±standard errors for height, weight and BMI in boys and girls (8–15 years) by year of evaluation, and results of pairwise comparisons.

	1992	1999	2012			
	Mean±SE	Mean±SE	Mean±SE	*F*	*p-*value	Pairwise comparisons
**Boys**						
Height	142.5±0.4	146.4±0.3	145.3±0.3	30.6	<0.001	92<99;92<12;99>12
Weight	33.7±0.4	35.7±0.2	38.1±0.3	47.6	<0.001	92<99<12
BMI	16.2±0.1	16.3±0.1	17.7±0.1	69.7	<0.001	92 = 99;92<12;99<12
**Girls**						
Height	144.0±0.5	147.1±0.3	144.2±0.3	33.6	<0.001	92<99;92 = 12;99>12
Weight	38.3±0.5	39.3±0.3	38.8±0.3	1.9	0.146	92 = 99 = 12
BMI	17.9±0.2	17.7±0.1	18.4±0.1	8.0	<0.001	92 = 99;92 = 12;99<12

**Figure 1:**
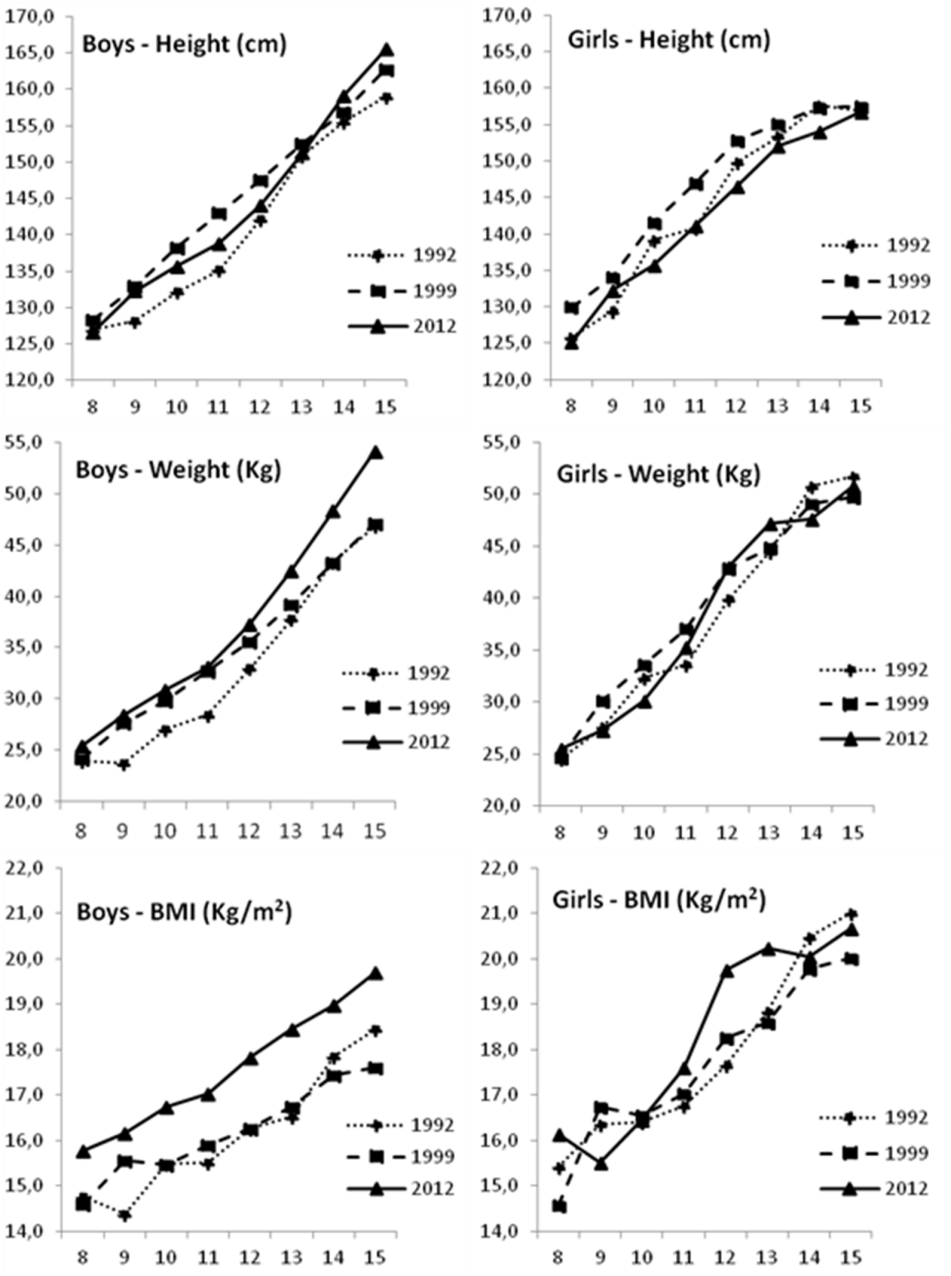
Secular trends for stature, body weight and BMI by age and sex, adjusted for biological maturation (1992, 1999, 2012).

Table 4 shows ANCOVA results for the mean differences across years for each variable by sex, for each age, adjusted for biological maturation. In height, except for ages 8 and 13 in boys, and 15 year old girls, significant differences (p<0.05) were found in both sexes. For weight, differences were observed at ages 9–15 years in boys, and at ages 10–11 in girls (p<0.05). For BMI, significant differences (p<0.05) were noticed at all ages for boys, whereas for girls differences were only noticed at ages 12–13 years. In summary, increases in weight and BMI were observed for each age from 1992 to 2012 in boys, with those from 2012 been, in general, heavier and having the highest BMI; among girls, those from 1999 were heavier (ages 10 and 11), and those from 2012 reported the highest BMI (ages 12 and 13); however, in both sexes, no significant difference was found from 1992 to 1999 for BMI (in all ages) and weight (except for age 9 in boys, and age 11 in boys and girls). With respect to height, generally, children from 1999 reported the highest values, but comparing values from 1992 to those from 2012, an increase in stature was noticed in boys, but not in girls. Figure 1 shows the secular trends in stature, body weight and BMI along these three time points (1992, 1999 and 2012), by age and sex.

**Table 4:** Biological maturation-adjusted means±standard errors for height (cm), weight (kg) and BMI (kg/m^2^) in boys and girls by age and study years (ANCOVA results)

	Height				Weight				BMI			
	1992(M±SE)	1999(M±SE)	2012(M±SE)	Pairwisecomparisons(only whenF has a p<0,05)	1992(M±SE)	1999(M±SE)	2012(M±SE)	Pairwisecomparisons(only when F has a p<0,05)	1992(M±SE)	1999(M±SE)	2012(M±SE)	Pairwisecomparisons(only whenF has a p<0.05)
**Boys**												
8	127.0±1.2	128.3±0.9	126.7±0.7	-	23.9±0.8	24.1±0.6	25.5±0.5	-	14.8±0.3	14.6±0.2	15.8±0.2	92 = 99;92<12;99<12
9	128.1±1.5	132.9±0.9	132.3±0.8	92<99;92<12;99 = 12	23.7±1.1	27.6±0.7	28.4±0.6	92<99;92<12;99 = 12	14.4±0.5	15.6±0.3	16.2±0.2	92 = 99;92<12;99 = 12
10	132.1±1.0	138.3±0.9	135.6±0.6	92<99;92<12;99>12	27.1±1.0	29.8±0.8	30.9±0.6	92 = 99;92<12;99 = 12	15.5±0.4	15.5±0.4	16.7±0.2	92 = 99;92<12;99<12
11	135.1±1.1	143.0±0.9	138.8±0.8	92<99;92<12;99>12	28.5±1.0	32.7±0.7	33.1±0.7	92<99;92<12;99 = 12	15.5±0.3	15.9±0.3	17.0±0.2	92 = 99;92<12;99<12
12	142.1±1.2	147.6±0.7	144.0±0.8	92<99;92 = 12;99>12	33.0±1.1	35.6±0.6	37.3±0.7	92 = 99;92<12;99 = 12	16.3±0.4	16.3±0.2	17.8±0.2	92 = 99;92<12;99<12
13	150.8±1.0	152.5±0.5	151.2±0.8	-	37.8±1.0	39.2±0.5	42.5±0.8	92 = 99;92<12;99<12	16.5±0.3	16.7±0.2	18.4±0.3	92 = 99;92<12;99<12
14	155.5±1.5	156.9±0.6	159.1±0.7	92 = 99;92 = 12;99<12	43.3±1.8	43.2±0.7	48.3±0.8	92 = 99;92<12;99<12	17.8±0.6	17.5±0.2	19.0±0.3	92 = 99;92 = 12;99<12
15	159.0±1.4	162.7±0.8	165.5±1.0	92 = 99;92<12;99 = 12	46.8±1.5	47.0±0.9	54.1±1.2	92 = 99;92<12;99<12	18.5±0.4	17.6±0.2	19.7±0.3	92 = 99;92 = 12;99<12
**Girls**												
8	125.9±1.3	129.9±1.1	125.1±1.2	92<99;92 = 12;99 = 12	24.5±0.9	24.6±0.8	25.5±0.8	-	15.4±0.4	14.6±0.4	16.2±0.4	-
9	129.4±1.8	134.0±1.4	132.2±1.6	92<99;92 = 12;99 = 12	27.5±1.4	30.1±1.1	27.3±1.2	-	16.4±0.6	16.7±0.5	15.5±0.5	-
10	139.2±1.7	141.6±1.0	135.8±1.0	92 = 99;92 = 12;99>12	32.3±1.4	33.6±0.8	30.1±0.8	92 = 99;92 = 12;99>12	16.4±0.6	16.6±0.3	16.5±0.3	-
11	140.8±1.0	146.9±0.7	141.1±0.7	92<99;92 = 12;99>12	33.6±1.1	37.1±0.7	35.3±0.7	92<99;92 = 12;99 = 12	16.8±0.4	17±0.3	17.6±0.3	-
12	149.8±1.3	152.8±0.7	146.5±0.8	92 = 99;92 = 12;99>12	39.8±1.8	42.9±0.9	43.0±1.0	-	17.7±0.6	18.3±0.3	19.8±0.3	92 = 99;92<12;99<12
13	153.3±1.0	155.0±0.5	152.1±0.9	92 = 99;92 = 12;99>12	44.4±1.3	44.8±0.7	47.1±1.2	-	18.8±0.5	18.6±0.2	20.2±0.4	92 = 99;92 = 12;99<12
14	157.6±1.1	157.3±0.6	154.1±0.6	92 = 99;92>12;99>12	50.8±1.5	49.1±0.8	47.6±0.8	-	20.5±0.5	19.8±0.3	20.0±0.3	-
15	156.9±1.2	157.5±0.5	156.7±1.0	-	51.7±1.4	49.7±0.6	50.8±1.2	-	21±0.6	20±0.2	20.7±0.5	-

Changes in nutritional status across the three time periods are summarized in Table 5. In general, in the past 20 years, a positive trend was observed for the prevalence of overweight and obesity, whereas a negative trend was observed for stunting-wasting (for boys), and normal-weight (for girls) prevalence; regarding the wasting prevalence, an increase between 1992–1999, followed by a decrease between 1999–2012, was observed; no clear trend was evident for the stunting category.

**Table 5:** Prevalence of nutritional status by sex and years of evaluation, and differences in prevalence between the years of evaluation by sex.

		1992		1999		2012			
		N	%	N	%	N	%	trend	*p-value*
**Boys**	Normal	187	75.7	495	67.0	454	73.9	ns¥	0.611
	Stunted	15	6.1	31	4.2	36	5.9	ns¥	0.722
	Wasted	33	13.4	150	20.3	51	8.3	Negative	<0.001
	Stunted_Wasted	8	3.2	27	3.7	5	0.8	Negative	0.005
	Overweight	2	0.8	24	3.2	31	5.0	Positive	0.002
	Obese	2	0.8	12	1.6	37	6.0	Positive	<0.001
**Girls**	Normal	216	78.3	622	75.3	473	70.4	Negative	0.006
	Stunted	24	8.7	20	2.4	35	5.2	ns¥	0.273
	Wasted	16	5.8	88	10.7	25	3.7	Negative	0.009
	Stunted_Wasted	1	0.4	6	0.7	3	0.4	ns¥	0.916
	Overweight	14	5.1	53	6.4	75	11.2	Positive	0.001
	Obese	5	1.8	37	4.5	61	9.1	Positive	<0.001

¥, ns = non-significant.

## Discussion

The purpose of this study was to examine secular changes in growth and nutritional status in Mozambican children and adolescents between 1992, 1999 and 2012, which comprises a time from just after the War to a period of relevant economic growth and improvement in quality of life. In general, a positive secular trend was observed in height, weight and BMI mean values among Mozambican youth aged 8–15 years. This may reflect changes in population general improvements in their lifestyles, health, nutritional and economic conditions during this transitional period.

During the last century, positive secular trends in youths’ and adults’ statures and weights have been consistently observed all over the world [4], although they vary among populations [10], [12]–[19]. For example, Garcia and Quintana-Domique [14] studied height trends among 10 European countries and found increases in adult subjects from 1950 to 1980. Similarly, Danker-Hopfe and Roczen [13] reported a positive trend in stature of German children from 1968 to 1987. On the other hand a negative secular trend in the height of women from Kenya and Senegal was reported [17], whereas Zellne et al [15] and Krawczynski et al [16] found a plateau in German and Polish children’s height, respectively. Additionally, positive changes have also been observed in weight all over the world reflecting recent increases in the prevalence of overweight/obesity in children [10]. The overall positive secular trends in human physical growth found in Mozambican children and youth of both sexes from 1992 to 2012 can be generally explained with the same reasons given in the previous studies. During this twenty-year period, Mozambique went through considerable social and economic transition, namely better nutritional factors, reduction of infectious diseases, improved access to the national health care system, and extended use of safe water, resulting in a highly favorable environment which translates to the positive secular trend. These changes, however, did not reach all subjects in the same way, and a stabilization, or even a decrease in girls’ height was observed, suggesting that during the first decade the social and economic transition in Mozambique had a positive impact on girls’ height; on the contrary, in the second decade the impact of this transition period was not positive anymore, providing some stabilization in girls’ growth.

Although Mozambican youth, namely boys, had become taller during the study period (1992–2012), stature increments were not always linear. This non-linearity in stature increments observed in secular changes studies was previously reported [31], and environmental constraints were related to them. Mozambican boys from 2012 were, in general, taller than those from 1992, but shorter than those from 1999, meaning that a decrease in mean stature occurred from 1999 to 2012 (see Table 3); among girls, those from 1999 were the tallest, and a significant decline or stabilization were observed from 1999 to 2012, or even from 1992 to 2012. Since the end of the War, Mozambique witnessed a systematic migration from rural cities to the capital city of Maputo, thereby increasing the amount of urban population as well as social inequalities [32], [33]. For example, from 1997 to 2003, in Maputo, due to the high migration rate, the poverty rate increased from 67% to 70% [34]. These migrants, coming from rural areas live mostly in suburban regions, not in the Maputo central area; they live under precarious conditions, with negative impacts on children’s growth. So, when we stratified our sample into two groups (see Table 6), namely children living in the center of Maputo (urban area) and far from the center (suburban area, but still in Maputo city), no significant negative changes were observed in the height of urban boys, but significant negative changes in suburban boys’ heights from 1999 to 2012 were observed, and these values were responsible for the decline in boys’ mean stature observed in this period. Regarding girls, significant decreases were observed for urban girls’ heights from 1992–2012 and 1999–2012, and also significant negative changes in suburban girls’ height from 1999 to 2012; these results, together, could explain the absence of a clear trend in girls’ stature during the study period. Thus, during the first years after the end of the War, social and economic changes provided increments in the Mozambican quality of life, resulting in mean increments in children’s height. The high migration rate observed during the end of the first decade, and early of the second decade after the War, may have induced the decrease found in mean heights due to the fact that children from rural areas, that started living in the suburban area, were generally shorter than those born and living in the center of urban area. This means that the mean height decrease found from 1999 to 2012 can be associated to this migration process.

**Table 6:** Age and biological maturation-adjusted means±standard errors of boys and girls heights according to study year and region.

	1992		1999		2012				
	Mean±SE		Mean±SE		Mean±SE		*F*	*p-*value	Pairwise comparisons
**Urban**									
Boys	145.4±0.8		147.5±0.4		146.7±0.4		2.715	0.067	92 = 99 = 12
Girls	147.7±0.9		147.1±0.4		144.6±0.5		8.454	<0.001	92 = 99;92>12;99>12
**Suburban**									
Boys	141.5±0.5		145.6±0.3		143.7±0.4		27.213	<0.001	92<99;92<12;99>12
Girls	142.8±0.5		147.1±0.3		143.6±0.4		37.645	<0.001	92<99;92 = 12;99>12

In developing countries, the co-existence of under- and over-nutrition may be expected, especially if a high prevalence of malnourished children was observed in the past [35]. As we reported in Table 5, the prevalence of malnourished (stunted, wasted and stunted-wasted) children in 1992 in Mozambique was higher than the prevalence of over-nutrition (overweight/obese) –22.7% of boys and 14.9% of girls were malnourished, and 1.6% of boys and 6.9% of girls were overweight/obese. However, in 2012, significant increments were observed in the prevalence of overweight/obesity in children–11.1% of boys and 20.2% of girls were classified as overweight/obese, with a slight decrease in the prevalence of under-nutrition (15% for boys and 9.4% for girls). This emerging picture is similar to other developing countries where both nutritional conditions co-exist at the same time [36], [37]. By the fact that in the early 90′s Mozambique had a moderate prevalence of stunting (6.1% among boys, 8.7% among girls) and wasting (13.4% among boys, 5.8% among girls) among youth, the height and weight increments observed in the last two decades may be related to the reduction of these adverse growth statuses and better youth health conditions. However, in the present study we only found a significant decline in the prevalence of wasting (see Table 5), which is associated with mean weight gains over the years. Despite the reduction in malnutrition, namely in the wasted condition, an increase in the prevalence of overweight and obesity among youth was also observed. This scenario is the net result of the new way of life of the Mozambican population (especially youth) linked to the fast urbanization process occurring in parallel with the economic, social and cultural transitional period, characterized also by increments in fast-food consumption and decreases in health and traditional food consumption [38]. This factor, in association with a decrease in youth habitual physical activity levels, and an increase in time spend in sedentary activities [23], may be associated with mean weight increments, leading also to a reduction in the malnourished condition. Of course that at the same time the prevalence of overweight/obesity in children and adolescents has increased significantly.

Notwithstanding the relevance of the present results, this study has several limitations. Firstly, no direct information about the children’s nutritional habits and/or objectively measured physical activity levels and patterns were available which could be very helpful in explaining the results, namely the mean weight increases and changes on nutritional status prevalence. It has to be acknowledged that collecting this information in a systematic and objective way during the last 20 years would be a very daunting task. Secondly, as there is no general consensus about BMI cut-points for African children, the cut-points used may have overestimated/underestimated the malnourished and over-nutrition prevalence in youth. Thirdly, as the sample age is limited to 8–15 years, this did not allow us to observe if these trends exist in adults. Despite these limitations, this study also has significant merits: (1) the use of three time periods covering a relevant transitional period in Mozambique economic and social context; (2) the use of a consistent standard methodology to measure all children, having the same protocol in all time periods; (3) this is a study conducted in an emerging developing country, from the African continent, with almost no information regarding a most relevant issue – anthropometry as a well fashioned mirror of societal changes and their links to children and youth health.

## Conclusions

This study showed a positive trend in human physical growth in Mozambican children and youth between 1992 and 2012 which is related to economic, social and cultural changes occurring since the end of the War. Furthermore, these changes express a dual burden in this population. On one side we see a reduction in malnourished youth; on the other, a rise in the prevalence of overweight and obesity probably linked to the rapid urbanization process and lifestyle modifications. Public education policies and intervention programs must be established and implemented to stop the increase in overweight and obesity, which may reduce the risk of chronic diseases development in later life. Further, great care must be taken to reduce, or better eradicate the problem of under-nutrition in youth. Since urbanization sprawl and organization, food intake, levels of physical activity, and time spent in sedentary activities may also be associated with the present findings, future studies should be conducted to identify their impact on the growth and health of children and adolescents.
